# Importance of the residue 190 on bactericidal activity of the bactericidal/permeability-increasing protein 5

**DOI:** 10.18632/oncotarget.9292

**Published:** 2016-05-11

**Authors:** Hanwei Wu, Lu Liu, Muqi Lin, Li Liu, Chen He, Duo Zheng, Weiren Huang

**Affiliations:** ^1^ Key Laboratory of Medical Reprogramming Technology, Shenzhen Second People's Hospital, The First Affiliated Hospital of Shenzhen University, Shenzhen, China; ^2^ Department of Cell Biology and Genetics, School of Basic Medical Sciences, Shenzhen University Health Sciences Center, Shenzhen, China

**Keywords:** BPI, mutant, structural model, charged residue

## Abstract

The bactericidal/permeability-increasing protein (BPI) with bactericidal and endotoxin-neutralizing activity is of considerable interest in clinical applications. However, the crucial residues responsible for the bactericidal activity of BPI remain elusive. In previous study, we identified the mutation of mBPI5 associated with the male infertility of mice. Here, the effects of Asp190Ala mutation on the antibacterial activity of mBPI5 have been determined. Substitution of Asp190 by alanine caused significant improvement in cytotoxic effect toward both *E.coli* J5 and *P.aeruginosa*. Liposome co-sedimentation assay showed that the ratio of Asp190Ala mutant binding to lipids increased by 8 folds. These results were well consistent with known fact that antibacterial activity of BPI is attributed to its high affinity for lipid moiety of lipopolysaccharides (LPS). The constructed structure of mBPI5 revealed that Asp190 was located close to 6 positively charged residues on the surface of N-terminal domain. When replacing Asp190 with alanine, salt linkages with Arg188 were broken, making the side chain of Arg188 be free to move and form tighter contacts with negatively charged LPS. These findings suggest that residue 190 combined with surrounding positively charged residues largely contribute to bactericidal and endotoxin-neutralizing activities of mBPI5.

## INTRODUCTION

Gram-negative bacterial infections can result in severe pathologic changes including fever, disseminated intravascular coagulation and multisystem organ failure [1, 2]. The major virulence factor of the Gram-negative bacteria has been identified as lipopolysaccharides (LPS), a glycolipid structural component on the bacterial cell wall [3–5]. Release of LPS into the circulation induces a systemic inflammatory response [6]. Therefore, a promising therapeutic agent for Gram-negative bacteria infections has both antibacterial and antiendotoxic properties.

The bactericidal/permeability-increasing protein (BPI), a multifunctional cationic protein with 55 to 60 kDa, is originally found in granules of polymorphonuclear leukocytes, and more recently, is detected on the surface of neutrophils and in eosinophils [7–9]. Extensive studies show that BPI exhibits powerful antibacterial activity, largely due to the high affinity of BPI for the conserved lipid moiety in LPS [7, 10]. Binding of BPI to Gram-negative bacteria would initiate growth arrest, increase outer membrane permeability, selectively activate endogenous bacterial phospholipases and ultimately cause bacterial death [11, 12]. BPI also forms complex with cell-free LPS and consequently inhibits all LPS-induced host immune responses. Therefore, the antibacterial and antiendotoxin activities of BPI attract high interesting for the treatment of Gram-negative bacteria infections [13, 14].

Structural characterization of BPI has provided insight into its function. The crystal structures of human BPI show a boomerang-shaped molecule formed by two similar domains, N-terminal domain and C-terminal domain [15, 16]. A high proportion of basic residues are located at the surface and forms positively charged tips on N-terminal domain, resulting in high affinity of BPI with negatively charged LPS. This character is well consistent with observations that N-terminal region carries all the antibacterial and almost endotoxin-neutralizing activities of BPI [17–19]. For C-terminal domain, it is essentially neutral and only shows limited LPS-binding ability [20]. Few effects, however, have been directed toward targeting the precise residues or regions of BPI, where they contribute critically to the bactericidal and endotoxin neutralization actions.

Recently, we identified a putative causative mutation, replacing Asp190 with alanine in the mBPI5 gene (GenBank Accession No. NM_144890) that might be responsible for the male infertility of Blind sterile (bs) mice (data not shown). In this report, we extend our studies to investigate the effect of Asp190Ala mutation in mBPI5 on its antibacterial activity. Wild-type and this mutate of mBPI5 were expressed in *E. coli* as inclusion bodies, refolded by high concentration of urea and purified using His-tag. Antibacterial assay and liposome binding assay were performed to measure their corresponding changes caused by introduction of alanine on position 190. A structural model of mBPI5 was constructed to explore the mechanism underlying these changes.

## RESULTS

### The expression and purification of mBPI5 and Asp190Ala mutant

**Figure 1:**
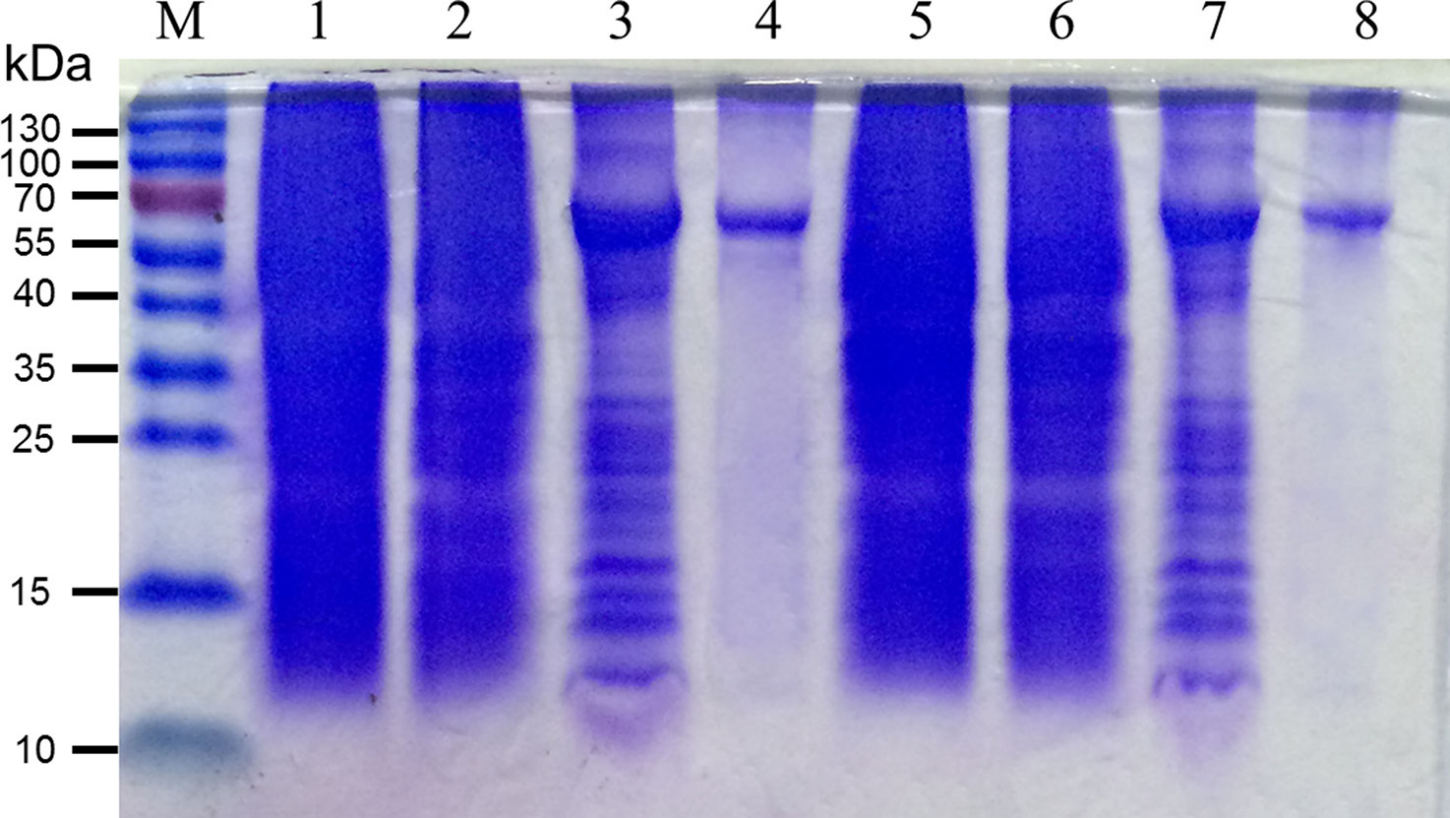
Production and characterization of mBPI5 and Asp190Ala mutant Lane M: markers; lane 1 and 5: whole cell lysates of mBPI5 and Asp190Ala mutant before IPTG induction; lane 2 and 6: sediment of mBPI5 and Asp190Ala mutant before IPTG induction; lane 3 and 7: sediment of mBPI5 and Asp190Ala mutant after IPTG induction; lanes 4 and 8: purified mBPI5 and Asp190Ala mutant.

Of great interests as novel antibiotics, large quantities of highly purified BPI are required to meet the needs of basic research and clinical trials. Among the systems available for heterologous protein production, *E. coli* has been the most widely used host. The coding sequences of mBPI5 and Asp190Ala mutant were cloned into the expression plasmid pET32a. mBPI5 and its mutant produced in *E. coli* strain SHuffle were expressed as inclusion bodies such that its lethal effect towards the host could be masked and protect them from proteolytic degradation. Inclusion bodies of mBPI5 were solubilized by the use of a high concentration of urea (8M) along with β-mercaptoethanol, yielding an appreciably high recovery of 26%. High quality purification of mBPI5 and Asp190Ala mutant could be achieved by one-step nickel-affinity chromatography (Figure 1).

### The effect of Asp190Ala mutation on antibacterial activity of BPI

BPI has potent bactericidal activity against a wide variety of Gram-negative organisms. Two representative Gram-negative clinical isolates, *E.coli J5 and P.aeruginosa* that have high intrinsic resistance to antibiotics, were chose to assess the effect of Asp190Ala mutation on the antibacterial activity [25].

**Figure 2:**
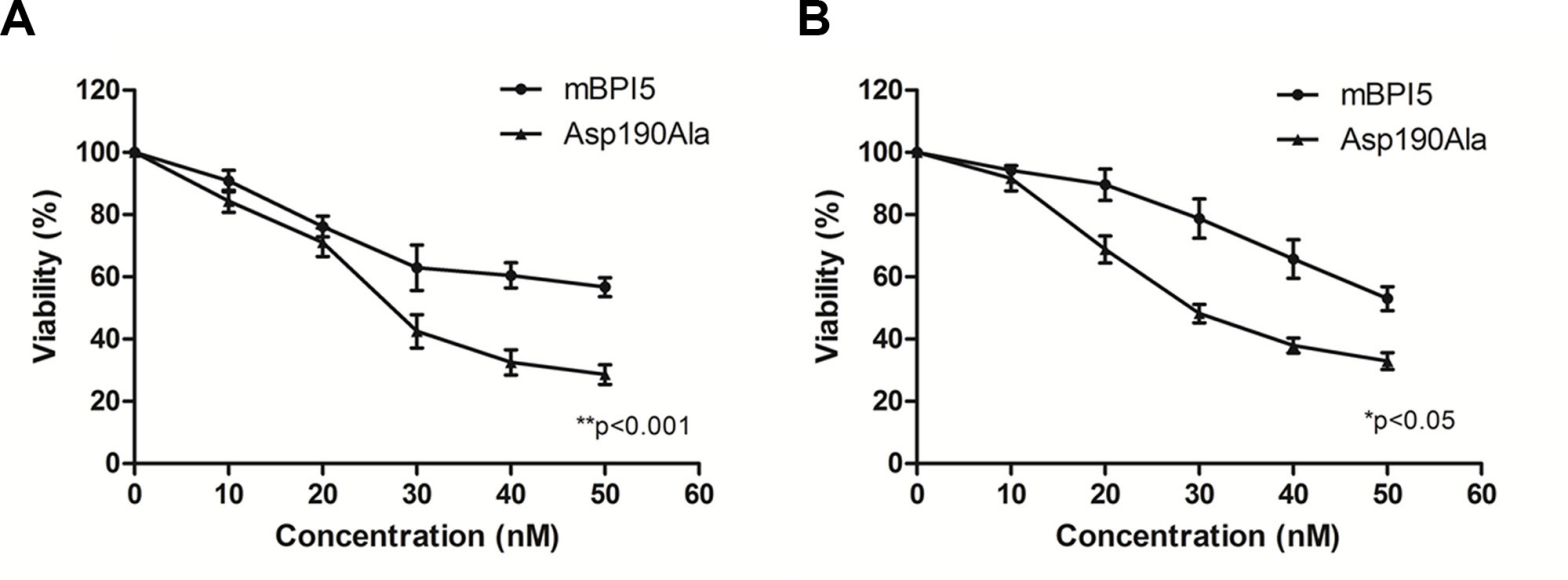
Comparison of bactericidal activity of mBPI5 and its Asp190Ala mutant toward *E.coli J5 and P.aeruginosa.* Both mBPI5 and Asp190Ala mutant can suppress *E. coli* J5 and *P.aeruginosa* in a concentration-dependent manner. Under relatively low concentrations (< 10 nM), the antibacterial activity of Asp190Ala mutant against two Gram-negative isolates is similar to that of wild-type. However, Asp190Ala mutant has a stronger effect on suppressing Gram-negative bacteria growth at higher concentrations. All data are shown as mean ± SD (*p < 0.05; **p < 0.001); bar SD.

As shown in Figure 2, both mBPI5 and Asp190Ala mutant suppressed the growth of *E.coli J5 and P.aeruginosa* in a concentration-dependent manner. Under relatively low concentrations (< 10 nM), the antibacterial activity of Asp190Ala mutant against two Gram-negative isolates was similar to that of wild-type. However, compared with wild-type mBPI, Asp190Ala mutant had a stronger effect on suppressing Gram-negative bacteria growth at higher concentrations. These results suggested that the introduction of alanine at residue 190 significantly enhanced the antibacterial activity of mBPI5. (Figure 2)

### The effect of Asp190Ala mutation on the binding affinity of mBPI5 to liposome

BPI's cytotoxic activity that is selectively manifest toward Gram-negative bacteria has been attributed to its high affinity for the lipid moiety of LPS or endotoxin [7, 10]. The negatively charged liposome has been used frequently for mimicking the negatively charged bacterial membrane [26]. The effect of Asp190Ala mutation on binding affinity was measured by using affinity precipitation assay.

**Figure 3:**
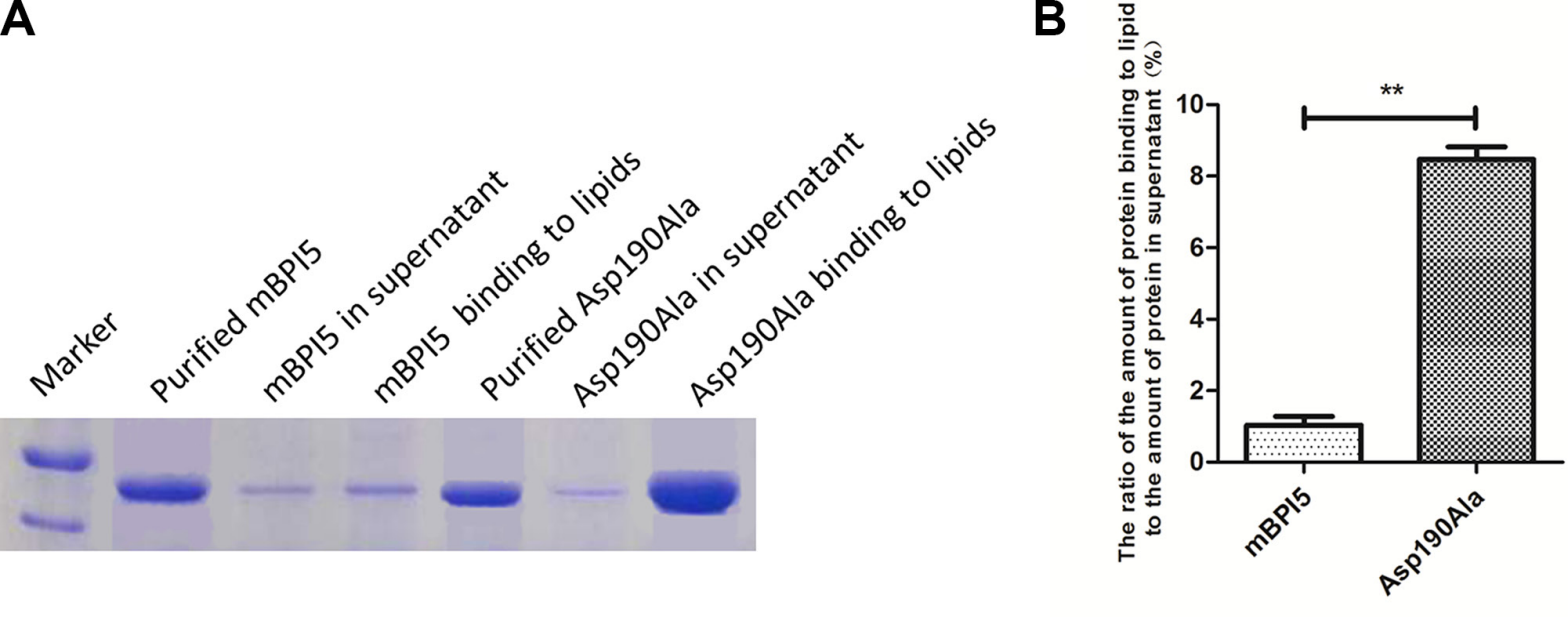
Comparison of the binding affinity of mBPI5 and Asp190Ala mutant to lipids (**A**) Asp190Ala mutant almost exists in the form of bound with lipid, whereas native mBPI5 binds weakly to liposomes. (**B**) The ratio of the amount of Asp190Ala mutant binding to lipid to the amount of it in supernatant increases about 8 folds compared with that of mBPI5. All data are shown as mean ± SD (*p < 0.05; **p < 0.001); bar SD.

As shown in Figure 3, Asp190Ala mutant almost existed in the form of bound with lipid, whereas native mBPI5 bound weakly to liposomes. The ratio of the amount of protein binding to liposome to the amount of protein in supernatant was 1.03 and 8.46 for mBPI5 and Asp190Ala mutant, respectively, that is, this ratio of Asp190Ala mutant increased about 8 folds compared with that of mBPI5. These data suggested that the enhanced binding affinity to lipid accounted for the increased antibacterial activity of Asp190Ala mutant. (Figure 3).

### The structural analysis of mBPI5

In order to shed light on mechanism by which introduction of alanine at position 190 greatly enhanced its antibacterial activity and affinity for lipid, a structural model of mBPI5 was required. Since no suitable structure templates that shared high sequence similarity with mBPI5 could be found, threading method (I-TASSER) was used to predict its structure, by which a library of representative structures was searched for structure analogs to mBPI5, mBPI5 was aligned with structure templates by secondary-structure enhanced profile-profile threading alignment, and structure models of mBPI5 were generated by iterative structural assembly simulations. The best model was selected based on its C-score, TM score and RMSD value.

**Figure 4:**
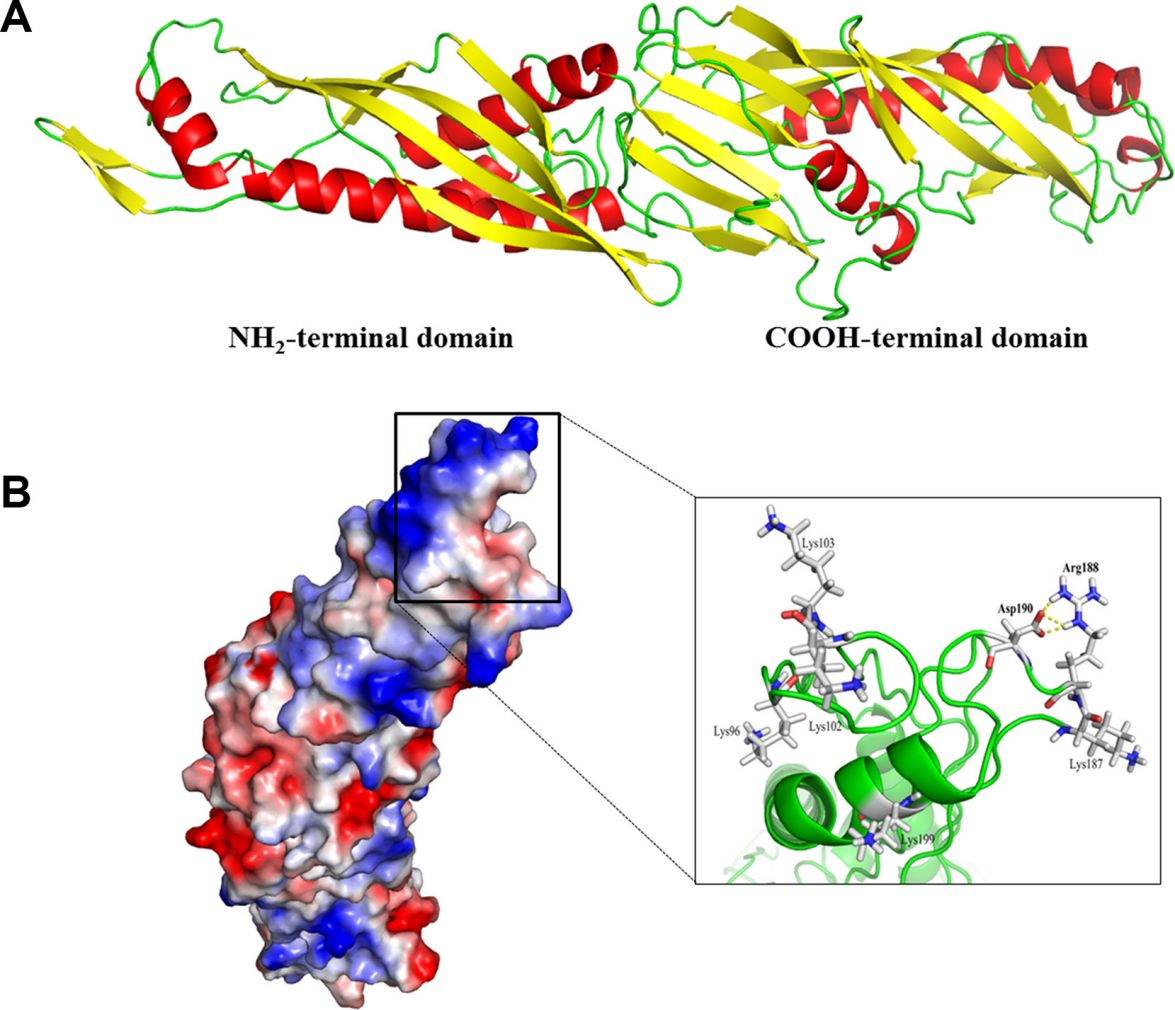
The overall structure of mBPI5 (**A**) The structure of mBPI5 constructed by I-TASSER. mBPI5 shows a boomerang-shaped molecule formed by two domains, N-terminal and C-terminal domain. (**B**) Surface representation of mBPI5 according to the electrostatic character of residues. The negatively charged carboxylate of Asp 190 forms salt linkages with the positively charged guanidinium at the end of the side chain of arginine 188. Six positively charged residues, including Lys96, Lys102, Lys103, Lys187, Arg188 and Lys199, fold into the proximity of Asp 190.

Just like that of human BPI, the dimensional structure of mBPI5 displayed boomerang-shaped molecule formed by two domains (Figure 4A). The N-terminal and C-terminal domain shared a similar two-layer α/β structure, a unique fold only found in BPI family. Asp190 was located on the surface of the N-terminal domain. Closer examination showed that the negatively charged carboxylate of Asp190 formed salt linkages with the positively charged guanidinium at the end of the side chain of arginine 188 (Figure 4B). When replacing Asp190 with alanine, these electrostatic interactions were broken so that the positively charged side chain of Arg188 was free to move and established tighter contacts with negatively charged LPS.

Besides Arg188, other five basic residues, including Lys96, Lys102, Lys103, Lys187 and Lys199, were folded into the proximity of Asp190, that is, a high proportion of basic residues was concentrated in the vicinity of Asp190 (Figure 4B). Therefore, the presence of negatively charged Asp190 could lower and disturb electrostatic interactions between these basic residues and LPS.

## DISCUSSION

Although most Gram-negative bacteria are susceptible to antibiotics, therapies are needed to counteract the inflammatory effects of killed bacteria and/or their endotoxins [6]. Among the potential therapeutic agents for Gram-negative sepsis, BPI is especially promising since it has both antibacterial and antiendotoxic properties. In this study, we define the critical role of residue 190 in antibacterial and antiendotoxic activities. We found that the replacement of Asp190 with alanine can greatly enhance the antibacterial activity of mBPI5 to *E.coli* J5 and *P. aeruginosa*. The enhanced antibacterial activity of Asp190Ala mutant is brought about by a marked increase in affinity to lipids. Constructed structure of mBPI5 suggested that replacing Asp190 with alanine could avoid the unfavorable contacts of negatively charged Asp190 with negatively charged LPS. These results could improve our understanding of the structure-function relationship of BPI, which paves way for site-directed mutagenesis experiments to modify BPI for clinical applications.

## MATERIALS AND METHODS

### Gene cloning

Total RNA was extracted from the testis of mice using TRIzol reagent (Invitrogen, #10296028) and they were used as template for cDNA synthesis with RT Master Mix (TaKaRa, #RR036A). mBPI5 cDNA was amplified with the forward primer (5′-GACGA CGACAAGATGCTGCTGCTGTGGGCACT-3′) and the reverse primer (5′-GAGGAGAAGCCCGGTTACTCCG GAACGGTCAGCAG-3′). The resulting PCR product was cloned into the pET32a vector with a C-terminal His-tag. Successfully constructed plasmids were further confirmed by DNA sequencing. The Asp190Ala mutant was generated using a QuikChange™ site-directed mutagenesis kit (Stratagene, #200518) with the forward primer (5′-GAAGCGATCAGCCACCCT GTTGC-3′) and the reverse primer (5′-GCAACAGGGTG GCTGATCGCTTC-3′).

### Protein expression, refolding and purification

The recombinant mBPI5 and its mutant were expressed in *E. coli* strain SHuffle. Cells were grown at 37°C in LB medium containing 50 μg/ml Ampicillin to an OD600 of 0.6, and were then induced by the addition of 0.1 mM isopropyl β-D- thiogalactoside (IPTG) at 16°C for 12 h. Cells were harvested and re-suspended in buffer (25 mMTris-HCl pH 8.0, 300 mM NaCl), disrupted by sonication and centrifuged at 12000 g for 20 min at 4°C. Recombinant protein was exclusively detected in the precipitates. BPIs inclusion bodies were resuspended in 20 ml solubilization buffer (50 mM NaH_2_PO_4_, 300 mM NaCl, 10 mM imidazole, 8 M urea, pH 8.0), incubated by stirring for 2 h at room temperature, and then centrifuged at 12000 g for 20 min. The supernatant was applied to a nickel affinity column(Qiagen WorkBeads^™^ 40 Ni #40650003) equilibrated with the same buffer. Removal of contaminating proteins and initial refolding were carried out on the column by exchanging to nondenaturing buffer conditions with a linear 8–0 M urea gradient before elution of the bound protein. The recombinant BPIs was eluted with elution buffer (50 mM NaH_2_PO_4_, 300 mM NaCl, 300 mM imidazole, pH 8.0) and concentrated by ultrafiltration (Amicon #UFC500308), The eluted products were determined by Bradford protein assays (Bio-Rad Protein Assays Kits #5000001) analyzed by 15% reduced and non-reduced SDS-PAGE.

### Bactericidal activity assay

Cells were grown overnight in LB medium and then in galactose-free triethanolamine-buffered medium at 5 × 10^8^ to 10 × 10^8^ cells per mL corresponding to mid- to late-logarithmic phase. The culture was harvested and adjusted to an OD600 of 0.5 in physiological saline. The bacterial suspension (5 × 10^6^ cells) was incubated for 30 min at 37°C with various concentrations of mBPI5, Asp190Ala mutant or buffer control in a medium consisting of 10% Hanks balanced salt solution (GIBCO #14170112), 40 mM Tris-HCl pH 7.5, and 0.1% Casamino Acids in a total volume of 200 μL. MgCl_2_ was added, when desired, at a final concentration of 100 mM to the mixture described above. The effect of mBPI5 on bacterial viability was assessed by plating dilutions of the cells on nutrient agar plates and enumerating bacterial colonies after overnight incubation at 37°C.

### Liposome co-sedimentation assay

Liposome co-sedimentation assay was performed as described previously [21]. Lipid mixes (Avanti Polar Lipids) were suspended at 1 mg/ml in buffer containing 25 mM Hepes pH 7.4, 100 mM NaCl and 1 mM dithiothreitol, and liposomes were formed by sonication followed by hydration. Purified proteins were incubated with liposomes for 15 min at room temperature and were centrifuged at 60000 rpm for 20 min at 25°C. Obtained supernatants and pellets were subject to SDS-PAGE. The ratio of the amount of protein binding to lipid to the amount of protein in supernatants was determined using the Bradford protein assays (Bio-Rad Protein Assays Kits #5000001).

### Structure prediction of mBPI5

Since the crystal structure of mBPI5 was not available in PDB, to obtain the 3D structure of mBPI5, its sequence was submitted to online server, I-TASSER (Iterative Threading ASSEmbly Refinement), for sequence alignment and homology modeling. I-TASSER is an advanced protein homology algorithm, which uses multiple individual programs and steps as well as molecular dynamics to create protein structural models of submitted protein sequences [22, 23]. Models of mBPI5 created by I-TASSER were structurally evaluated using Verify3D [24]. The best 3D model was selected according to rank based on the C-score, TM score and RMSD value.

### Statistical Methods

#### Statistical analysis

All data were presented as mean ± standard deviation (SD). All statistical analyses were executed by using SPSS 17.0 software (IBM, Chicago, IL, USA). A two-sided value of *p < 0.05, **p < 0.001 was considered to be statistically significant.

## References

[1] Duma RJ (1985). Gram-negative bacillary infections. pathogenic and pathophysiologic correlates. Am J Med.

[2] Dellinger RP (2003). Inflammation and coagulation. implications for the septic patient. Clin Infect Dis.

[3] Alexander C, Rietschel ET (2001). Bacterial lipopolysaccharides and innate immunity. J Endotoxin Res.

[4] Rietschel ET, Kirikae T, Schade FU, Mamat U, Schmidt G, Loppnow H, Ulmer AJ, Zähringer U, Seydel U, Di Padova F, Max Schreier, Helmut Brade (1994). Bacterial endotoxin: molecular relationships of structure to activity and function. FASEB J.

[5] Beutler B, Rietschel ET (2003). Innate immune sensing and its roots. the story of endotoxin. Nat Rev Immunol.

[6] Mayeux PR (1997). Pathobiology of lipopolysaccharide. J Toxicol Environ Health.

[7] Weiss J, Elsbach P, Olsson I, Odeberg H (1978). Purification and characterization of a potent bactericidal and membrane active protein from the granules of human polymorphonuclear leukocytes. J Biol Chem.

[8] Calafat J, Janssen H, Tool A, Dentener MA, Knol EF, Rosenberg HF, Egesten A (1998). The bactericidal/permeability-increasing protein (BPI) is present in specific granules of human eosinophils. Blood.

[9] Weersink AJ, van Kessel KP, van den Tol ME, van Strijp JA, Torensma R, Verhoef J, Elsbach P, Weiss J (1993). Human granulocytes express a 55-kDa lipopolysaccharide-binding protein on the cell surface that is identical to the bactericidal/permeability-increasing protein. J Immunol.

[10] Gazzano-Santoro H, Parent JB, Grinna L, Horwitz A, Parsons T, Theofan G, Elsbach P, Weiss J, Conlon PJ (1992). High-affinity binding of the bactericidal/permeability-increasing protein and a recombinant amino-terminal fragment to the lipid A region of lipopolysaccharide. Infect Immun.

[11] Mannion BA, Weiss J, Elsbach P (1990). Separation of sublethal and lethal effects of the bactericidal/permeability increasing protein on Escherichia coli. J Clin Invest.

[12] Elsbach P (1998). The bactericidal/permeability-increasing protein (BPI) in antibacterial host defense. J Leukoc Biol.

[13] Levy O (2002). Therapeutic potential of the bactericidal/permeability-increasing protein. Expert OpinInvestig Drugs.

[14] Giroir BP, Carroll SF, Scannon PJ (1998). Bactericidal/Permeability-Increasing Protein (BPI): Structure, Function, and Clinical Applications. Yearbook of Intensive Care and Emergency Medicine.

[15] Beamer LJ, Carroll SF, Eisenberg D (1997). Crystal structure of human BPI and two bound phospholipids at 2.4 angstrom resolution. Science.

[16] Kleiger G, Beamer LJ, Grothe R, Mallick P, Eisenberg D (2000). The 1.7 Å crystal structure of BPI. a study of how two dissimilar amino acid sequences can adopt the same fold. J Mol Biol.

[17] Ooi CE, Weiss J, Elsbach P, Frangione B, Mannion B (1987). A 25-kDa NH2-terminal fragment carries all the antibacterial activities of the human neutrophil 60-kDa bactericidal/permeability-increasing protein. J Biol Chem.

[18] Gazzano-Santoro H, Mészáros K, Birr C, Carroll SF, Theofan G, Horwitz AH, Lim E, Aberle S, Kasler H, Parent JB (1994). Competition between rBPI23, a recombinant fragment of bactericidal/permeability-increasing protein, and lipopolysaccharide (LPS)-binding protein for binding to LPS and gram-negative bacteria. Infect Immun.

[19] Weiss J, Elsbach P, Shu C, Castillo J, Grinna L, Horwitz A, Theofan G (1992). Human bactericidal/permeability-increasing protein and a recombinant NH2-terminal fragment cause killing of serum-resistant gram-negative bacteria in whole blood and inhibit tumor necrosis factor release induced by the bacteria. J Clin Invest.

[20] Iovine NM, Elsbach P, Weiss J (1997). An opsonic function of the neutrophil bactericidal/permeability-increasing protein depends on both its N- and C-terminal domains. Proc Natl AcadSci U S A.

[21] Tsujita K, Suetsugu S, Sasaki N, Furutani M, Oikawa T, Takenawa T (2006). Coordination between the actin cytoskeleton and membrane deformation by a novel membrane tubulation domain of PCH proteins is involved in endocytosis. J Cell Biol.

[22] Roy A, Kucukural A, Zhang Y (2010). I-TASSER: a unified platform for automated protein structure and function prediction. Nat Protoc.

[23] Zhang Y (2008). I-TASSER server for protein 3D structure prediction. BMC Bioinformatics.

[24] Eisenberg D, Lüthy R, Bowie JU (1997). VERIFY3D: assessment of protein models with three-dimensional profiles. Methods Enzymol.

[25] Lister Philip D., Wolter Daniel J., Hanson Nancy D. (2009). Antibacterial-Resistant Pseudomonas aeruginosa: Clinical Impact and Complex Regulation of Chromosomally Encoded Resistance Mechanisms. Clin Microbiol Rev.

[26] Lee MK, Cha L, Lee SH, Hahm KS (2002). Role of amino acid residues within the disulfide loop of thanatin, a potent antibiotic peptide. J Biochem Mol Biol.

